# The Effect of Fused 12-Membered Nickel Metallacrowns on DNA and their Antibacterial Activity

**DOI:** 10.1155/S1565363303000074

**Published:** 2003

**Authors:** I. Tsivikas, M. Alexiou, A. A. Pantazaki, C. Dendrinou-Samara, D. A. Kyriakidis, D. P. Kessissoglou

**Affiliations:** 1 Laboratory of Inorganic Chemistry Department of Chemistry Aristotle University of Thessaloniki Thessaloniki 54124 Greece; 2 Laboratory of Biochemistry Department of Chemistry Aristotle University of Thessaloniki Thessaloniki 54124 Greece

## Abstract

The synthesis, characterization and the biological study of a series of Ni(ll)_2_(carboxylato)_2_ [12- MC_Ni(II)N(shi)2(pko)2_-4][12-MC_Ni(ii)N(sh03(pko)_-4] (CH_3_OH)_3_(H_3_O) fused 12-membered metallacrowns with 10 metal ions and commercial available herbicides or anti-inflammatory drugs as carboxylato ligands are reported. All the compounds have a mixed ligand composition with salicylhydroxamic acid and di-2-pyridylketonoxime as chelate agents. The compounds construct metallacrown cores {[12-MC_Ni(n)N(sj02(pko)2_-4][12-MC_Ni(ll)N(shO3(pko)_-4]}^2+^ following the pattern [-Ni-O-N-]_4_. The neutral decanuclear [Ni(II)(A)]_2_[12-MC_Ni(II)N(shi)2(pko)2_-4][12-MC_Ni(II)N(pko)3(pko)_-4] fused metallacrown, consists of two [12-MC_M(ox)N(ligand)_-4] units the {Ni(ll)(A)[12-MC_Ni(II)N(shi)2(pko)2_-4]} and {Ni(II)(A)[12-MC_Ni(II)N(shi)3(pko)_-4]} with 1^+^ and 1^-^ charge, respectively. Each metallacrown unit has four ring Ni(II) ions and one additional encapsulated Ni(II) ion in planar arrangement. The anionic unit is bonded with cationic one creating binuclear moieties. The herbicide or antiiflammatory carboxylato ligands are bridging the central octahedral nickel atom with a ring metal ion in a bindetate fashion. The effect on DNA and their antibacterial activity was examined. The changes in the mobility can be attributed to the altered structures of the pDNA treated with Ni(II) complexes. Evaluating the data of the antibacterial activity of the compounds tested, we can conclude that nickel complexes present strong antibacterial activity.


### The Effect of Fused 12-Membered Nickel Metallacrowns on DNA and their Antibacterial Activity

I. Tsivikas[b], M. Alexiou[a], A. A. Pantazaki[b], C. Dendrinou-Samara[a], D. A. Kyriakidis[b*] and D. P. Kessissoglou[a*]

*[a]Laboratory of Inorganic Chemistry, Department of Chemistry, Aristotle University of Thessaloniki, Thessaloniki 54124, GREECE; [b]Laboratory of Biochemistry, Department of Chemistry, Aristotle University of Thessaloniki, Thessaloniki, 54124, Greece*

(Received: July 20, 2002; Accepted: October 25, 2002)

### SYNOPSIS

The synthesis, the spectroscopic characterization and the biological study of a series of fused 12-membered nickel metallacrowns accommodating herbicides alkanoato or anti-inflammatory carboxylato ligands are reported. Evaluating the data of the antibacterial activity we can conclude that nickel complexes present strong antibacterial activity.

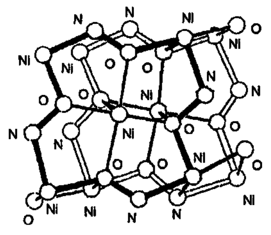

### ABSTRACT

The synthesis, characterization and the biological study of a series of $Ni(II)_2(carboxylato)_2$ [12-$MC_{Ni(II)N(shi)_2(pko)_2}$-4][12-$MC_{Ni(II)N(shi)_3(pko)}$-4] $(CH_3OH)_3(H_2O)$ fused 12-membered metallacrowns with 10 metal ions and commercial available herbicides or anti-inflammatory drugs as carboxylato ligands are reported. All the compounds have a mixed ligand composition with salicylhydroxamic acid and di-2-pyridyl-ketonoxime as chelate agents. The compounds construct metallacrown cores $\{$[12-$MC_{Ni(II)N(shi)2(pko)2}$-4][12-$MC_{Ni(II)N(shi)3(pko)}$-4]$\}^{2+}$ following the pattern $[\text{-Ni-O-N-}]_4$. The neutral decanuclear $[Ni(II)(A)]_2$[12-$MC_{Ni(II)N(shi)_2(pko)_2}$-4][12-$MC_{Ni(II)N(shi)_3(pko)}$-4] fused metallacrown, consists of two [12-$MC_{M(ox)N(ligand)}$-4]

units the {Ni(II)(A)[$12\text{-}MC_{Ni(II)N(shi)_2(pko)_2}$-4]} and {Ni(II)(A)[$12\text{-}MC_{Ni(II)N(shi)_3(pko)}$-4]} with $1^+$ and $1^-$ charge, respectively. Each metallacrown unit has four ring Ni(II) ions and one additional encapsulated Ni(II) ion in planar arrangement. The anionic unit is bonded with cationic one creating binuclear moieties. The herbicide or antiiflammatory carboxylato ligands are bridging the central octahedral nickel atom with a ring metal ion in a bindetate fashion. The effect on DNA and their antibacterial activity was examined. The changes in the mobility can be attributed to the altered structures of the pDNA treated with Ni(II) complexes. Evaluating the data of the antibacterial activity of the compounds tested, we can conclude that nickel complexes present strong antibacterial activity.

**Key words**: metallacrown, nickel, antibacterial activity.

### INTRODUCTION

Metallomacrocycles have gained increasing attention over the past decade due to their potentially unique properties. Metallacrowns are an example of this molecular class that exhibit selective recognition of cations and anions, can display intramolecular magnetic exchange interactions and may be used as building blocks for chiral layered solids /1/. Structurally, metallacrowns* resemble crown ethers in their repeating pattern of O-X-X-O with the oxygen atoms oriented toward the center of a cavity /2-27/. [$9\text{-}MC_{M(ox)N(ligand)}$ -3] /2-4/, [$12\text{-}MC_{M(ox)N(ligand)}$-4] /5-12/ and [$15\text{-}MC_{M(ox)N(ligand)}$-5] /13-18/ metallacrowns with cavity size 0.35 Å, 0.60 Å and 0.77 Å respectively and metal ions $Mn^{III}$, $Fe^{III}$, $Ni^{II}$, $Cu^{II}$ and $V^{V}O$, [12-MC-6] /26/, [16-MC-8] /27/, [18-MC-6] /19/, [18-MC-8] /26/, [30-MC-10] /20/ and stacking metallacrowns /21-23/ as well as a variety of dimers and fused metallacrowns /11,17,24,25/ have been reported.

Nickel compounds are speculated to be carcinogenic /28/ both in humans and experimental animals /29-33/. Because of the chemical nature of nickel, it is expected to form covalent bonds with DNA at several available binding sites such as nitrogen and oxygen centers of nucleobases and phosphate oxygens. Covalent $Ni^{2+}$ coordination to the N7 atom of guanine and adenine has been reported /34/ and this covalent interaction can be considered responsible for base depurination, predominantly at the adenine sites, resulting in extensive DNA damage /35/. Recent evidence suggests that DNA repair systems are very sensitive targets for Ni(II), resulting in a reduced removal of damaged DNA caused by environmental agents, which in turn may increase the risk of tumor formation.

We have initiated studies on the co-ordination chemistry of herbicide and/or anti-inflammatory carboxylate agents with Cu(II), Mn(II) and $d^{10}$ ions in an attempt to examine their mode of binding and their

---

* Metallacrown Nomenclature. The nomenclature for metallacrowns is as follows: $M'_mA_a[X\text{-}MC_{M(ox)H(Z)}\text{-}Y]$, where X and Y indicate ring size and number of oxygen donor atoms respectively, MC specifies a metallacrown, M and ox are the ring metal and its oxidation state, H is the identity of the remaining heteroatom bridge, and (Z) is an abbreviation for the organic ligand containing the N/O functionality. There are m captured metals (M') and a bridging anions (A) bound to the ring oxygens and metals, respectively.

biological behavior/36-43/. Recently, we have also reported the biological behavior of polynuclear nickel complexes with 3, 4 and 5 metal ions /44/.

Here we report the synthesis, characterization and the biological study of a series of $Ni(II)_2(carboxylato)_2$ $[12\text{-}MC_{Ni(II)N(shi)_2(pko)_2}\text{-}4][12\text{-}MC_{Ni(II)N(shi)_3(pko)}\text{-}4]$ $(CH_3OH)_3(H_2O)$ fused 12-membered metallacrowns with 10 metal ions and commercial available herbicides or anti-inflammatory drugs as carboxylato ligands (**Scheme 1**).

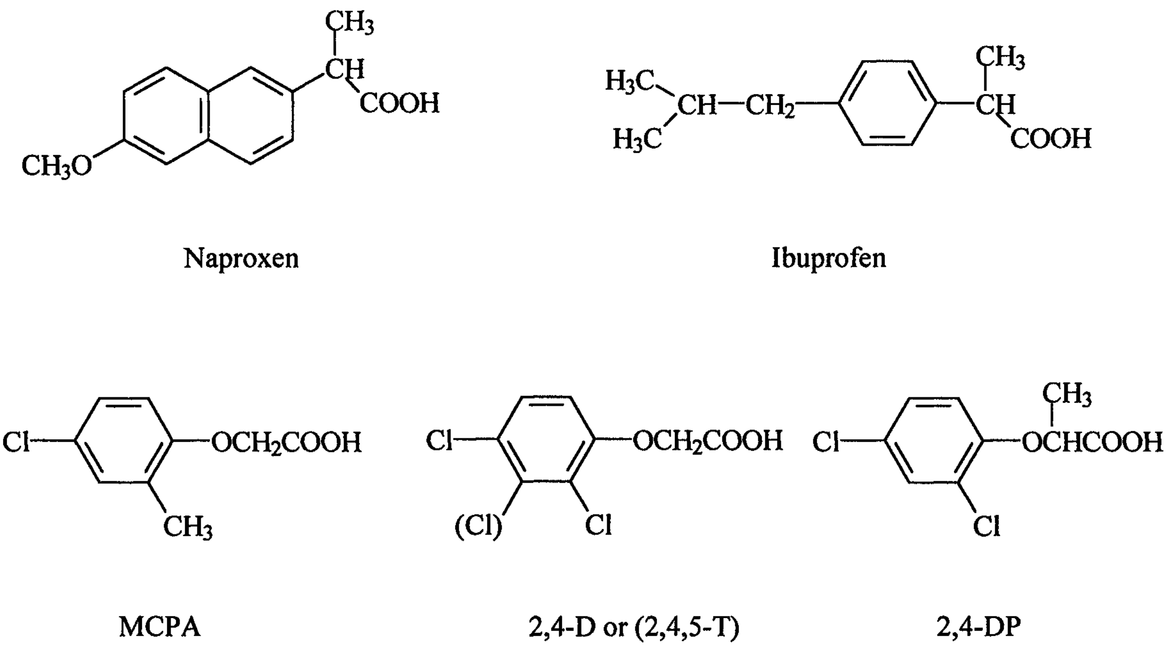

**Scheme 1.** The structural formulas of herbicides and anti-inflammatory drugs

### EXPERIMENTAL SECTION

### Abbreviations:

*H-2,4-D* = 2,4-dichlorophenoxy-acetic acid. *H-2,4,5,-T* = 2,4,5-trichlorophenoxyacetic acid. *HMCPA* = 2-methyl-4-chloro-phenoxyacetic acid). *H-2,4-DP* = 2,4-dichlorophenoxy-propionic acid. *naproxen* = *S*(+)-6-methoxy-α-methyl-2-naphthalene-acetic acid. *ibuprofen* = *S*(+)-4-isobutyl-α-methylphenylacetic acid. *$H_3shi$* = salicylhydroxamic acid. *Hpko* = di-2-pyridyl-ketonoxime.

### Materials

The chemicals for the synthesis of the compounds were used as purchased. Dimethylformamide (*dmf*) distilled from calcium hydride ($CaH_2$) and $CH_3OH$ from magnesium (Mg) were stored over 3Å molecular sieves. *H-2,4-D, H-2,4-DP, H-2,4,5,-T, HMCPA, naproxen, ibuprofen* and $NiCl_2 \cdot 6H_2O$ were purchased from Aldrich Co. All chemicals and solvents were reagent grade. Agarose was purchased from BRL. Tryptone and yeast extract were purchased from Oxoid (Unipath LTD, Hampshire, UK). Molecular weight markers, 1Kb DNA ladder, was from Gibco BRL. Plasmid pUC19 or pTZ18R was isolated from *E. coli XL1.*

### Culture media:

**MMS (Minimal Medium Salts broth):** 1.5% (w/v) glucose, 0.5% (w/v) $NH_4Cl$, 0.5% (w/v) $K_2HPO_4$, 0.1% (w/v) NaCl, 0.01% (w/v) $MgSO_4$ $7H_2O$ and 0.1% (w/v) yeast extract.

**Luria Broth Medium:** 1% (w/v) tryptone, 0.5% (w/v) NaCl and 0.5% (w/v) yeast extract. The pH of the media was adjusted to 7.0.

### Methods

Infrared spectra (200-4000 $cm^{-1}$) were recorded on a Perkin Elmer FT-IR 1650 spectrometer with samples prepared as KBr pellets. UV/VIS spectra were recorded on a Shimadzu-160A dual beam and on a Perkin-Elmer Lambda 9 UV/vis/near-IR spectrophotometer equipped with a Perkin-Elmer 3600 data station. $^1H$ NMR spectra of the complexes were obtained on a Bruker 200 MHz FT-NMR spectrometer operating in the quadrature detection mode ($^1H$ frequency, 200.1 MHz). Between 2000 and 5000 transients were accumulated over a 75 kHz bandwidth for each sample. The spectra contained 8000 data points, and the signal to noise ratio was improved by apodization of the free induction decay, which introduced a negligible 20 Hz line broadening. Baseline corrections of the NMR spectra were accomplished by a spline fir of baseline points chosen to minimize alteration of the peak line shape, position, and resolution. Chemical shifts were referenced to resonances due to residual protons present in the deuterated solvents. FAB mass spectra were acquired by the University of Michigan Mass Spectroscopy Facility. C, H and N elemental analysis were performed on a Perkin-Elmer 240B elemental analyser. Ni was determined by atomic absorption spectroscopy on a Perkin-Elmer 1100B spectrophotometer. Electric conductance measurements were carried out with a WTW model LF 530 conductivity outfit and a type C cell, which had a cell constant of 0.996. This represents a mean value calibrated at 25°C with potassium chloride. All temperatures were controlled with an accuracy of ± 0.1 °C using a Haake thermoelectric circulating system. All plastics and glassware used in the experiments with nucleic acids were autoclaved for 30 min at 120°C and 130 KPa. Heat-resistant solutions were similarly treated, while heat-sensitive reagents were sterilized by filter.

### Plasmid isolation:

Plasmids pUC19 and pTZ18R were isolated from *E. coli XL1* by the alkaline SDS lysis method (Strategene). Native DNA was isolated from calf thymus gland using standard procedure. Linear DNA resulted from incubation of the plasmid with the restriction enzyme EcoRI. Single stranded (ss) DNA was prepared by heating double stranded (ds) DNA at 100 °C for 10 minutes.

### Agarose gel electrophoresis of nucleic acids:

Aliquots of 1-3 μg of each nucleic acid (as indicated in the legends) were incubated in the presence of compounds 1-6 in a final volume of 20 μl. The reaction was incubated for 30 min at a constant temperature of 37 °C. It was terminated by the addition of 5 μl loading buffer consisting of 0.25% bromophenol blue, 0.25% xylene cyanol FF and 30% glycerol in water. The products resulting from DNA-compound interactions were separated by electrophoresis on agarose gels (1%), which contained 1μg/ml ethidium bromide in 40 mM Tris-acetate, pH 7.5, 20 mM sodium acetate, 2 mM $Na_2EDTA$ at 5 V/cm. Agarose gel electrophoresis was performed with a horizontal gel apparatus (Mini-Sub™ DNA Cell, BioRad) for around 4 h. Since ethidium bromide forms a fluorescent complex when it binds to DNA, a decreased of fluorescence signifies diminution of the amount of DNA. The gels were visualized in the presence of UV light. All assays were duplicated.

### Antibacterial activity:

The antibacterial activity of the compounds was studied against *B. substilis* (wild type), *B. cereus* (wild type), *S. aureus* (wild type), *E. coli* (XL1), *P. mirabilis* (wild type) and *X. campestris* (ATCC 33013). The screening was performed by the method of Minimal Inhibitory Concentration (MIC). Two different media (LB and MMS) were used. The compounds were dissolved in distilled water with a 2-fold successive serial dilution from 100 to 12 $\mu g.ml^{-1}$. All cultures were incubated at 37°C, except *X. campestris,* which was cultivated at 28 °C. Control tests with no active ingredients were also performed.

### Minimal Inhibitory Concentration (M.I.C.):

Minimal Inhibitory Concentration (M.I.C.) is determined using the method of progressive double dilution in liquid media containing 100 to 1 μg/ml of the compound being tested. A preculture of bacteria was grown in LB overnight at the optimal temperature of each species. 2ml of MMS were inoculated with 20μl of this preculture. This culture was used as control to examine if the growth of the bacteria tested is normal. In a similar second culture 20μl of the bacteria as well as the concentration of the compound tested was added. A third sample containing 2ml MMS supplemented with the same concentration of the compound tested was used as a second control to check the effect of the compound on MMS. All samples were in duplicate. We monitored the bacterial growth by measuring the turbidity of the culture in the tubes after 12 and 24 h. If a certain concentration of a compound inhibits bacterial growth we check the effect of the compound at the half concentration. This procedure continues until a concentration in which bacteria grow normally. The latest concentration that inhibits of bacterial growth is the M.I.C. value. All equipment and culture media are sterilized.

### Preparation of the Complexes:

The metallacrown compounds **2, 3, 4** with *2,4,5-T, MCPA,* and *2,4-D* as accommodated ligands respectively, were prepared according to the procedures reported previously /11,17,24/.

**[Ni(II)(2,4-DP)]$_2$[12-MC$_{Ni(II)N(shi)_2(pko)_2}$-4][12-MC$_{Ni(II)N(shi)_3(pko)}$-4](MeOH)$_3$(H$_2$O) (1):**

The sodium salts of *$H_3shi$* (0.765 g, 5 mmol), *Hpko* (0.597 g, 3 mmol) and $NiCl_2.6H_2O$ (2.37 g, 10 mmol) in 50 mL of freshly distilled methanol are dissolved. The reaction mixture was stirred for 1 h and an excess of the sodium salt of *2,4-DP* (1.41 g, 6 mmol) in methanol was added. Red/Brown microcrystalline product was obtained by slow evaporation of the mother liquid into 4 days. Yield 65%. Analytical data: (Fw = 2436.66) Found: C, 40.70; H, 4.50; N, 8.85; Ni, 23.55. $C_{82}H_{114}Cl_4N_{16}Ni_{10}O_{24}$ requires C, 40.42; H, 4.72; N, 9.20; Ni, 24.09); IR: $\nu_{max}/cm^{-1}$: (KBr pellet): $\nu(C{=}N)_{shi,pko}$:1599(vs); $\nu_{asym}(CO_2)_{2,4\text{-}DP}$: 1575(s); $\nu(C{=}O_{ph})_{shi}$: 1479(s), $\nu(C{=}O_{ox})_{shi}$: 1465(s); $\nu_{sym}(CO_2)_{2,4\text{-}DP}$: 1439(s); $\nu(N\text{-}O_{ox})_{shi,\ pko}$: 1258(s); UV-Vis: λ(nm) (ε, $dm^3.mol^{-1}.cm^{-1}$): *dmf* solution: 450(7260), 356(10310); *dmso* solution: 452(5130), 342(19930)

**[Ni(II)(naproxen)]$_2$[12-MC$_{Ni(II)N(shi)_2(pko)_2}$-4][12-MC$_{Ni(II)N(shi)_3(pko)}$-4](MeOH)$_3$(H$_2$O) (5):**

This compound was prepared in a similar way. The sodium salt of *S-[+]-naproxen* was used instead of the sodium salt *2,4-DP*. Yield 55%. Analytical data for $C_{99}H_{84}N_{14}Ni_{10}O_{28}$ (Fw = 2504.76) Found: C, 47.70; H, 3.20; N, 7.70; Ni, 22.75; requires C, 47.47; H, 3.38; N, 7.83; Ni, 23.43

IR: $\nu_{max}/cm^{-1}$: (KBr pellet): $\nu(C{=}N)_{shi,pko}$:1601(vs); $\nu_{asym}(CO_2)_{naproxen}$: 1572(s); $\nu(C{=}O_{ph})_{shi}$: 1482(s), $\nu(C{=}O_{ox})_{shi}$: 1465(s); $\nu_{sym}(CO_2)_{naproxen}$: 1438(s); $\nu$ $(N\text{-}O_{ox})_{shi,\ pko}$: 1264(s), UV-Vis: λ(nm) (ε, $dm^3.mol^{-1}.cm^{-1}$): *dmf* solution: 460(3415), 345(14965), 310(22200).

**[Ni(II)(ibuprofen)]$_2$[12-MC$_{Ni(II)N(shi)_2(pko)_2}$-4][12-MC$_{Ni(II)N(shi)_3(pko)}$-4](MeOH)$_3$(H$_2$O) (6):**

This compound was prepared in a similar way. The sodium salt of *S-[+]-ibuprofen* was used instead of the sodium salt *2,4-DP*. Yield 65%. Analytical data for $C_{97}H_{92}N_{14}Ni_{10}O_{26}$ (Fw = 2456.81) Found: C, 47.10; H, 3.50; N, 7.80; Ni, 23.05; requires C, 47.42; H, 3.77; N, 7.98; Ni, 23.89.

IR: $\nu_{max}/cm^{-1}$: (KBr pellet): $\nu(C{=}N)_{shi,pko}$:1599(vs); $\nu_{asym}(CO_2)_{ibuprofen}$: 1573(s); $\nu(C{=}O_{ph})_{shi}$: 1480(s), $\nu(C{=}O_{ox})_{shi}$: 1465(s); $\nu_{sym}(CO_2)_{ibuprofen}$: 1439(s); $\nu(N\text{-}O_{ox})_{shi,\ pko}$: 1263(s), UV-Vis: λ(nm) (ε, $dm^3.mol^{-1}.cm^{-1}$): *dmf* solution: 480(4800), 345(21833), FAB-MS (+)(*dmso* solution) molecular ion {[Ni(II)(ibuprofen)]$_2$[12-MC$_{Ni(II)N(shi)_2(pko)_2}$-4][12-MC$_{Ni(II)N(shi)_3(pko)}$-4]} at m/z:2341.

### RESULTS AND DISCUSSION

The synthesis of the nickel metallacrowns has been achieved via the reaction of $NiCl_2 6H_2O$ with the deprotonated salicylhydroxamic acid and ketonoximic acid followed by the addition of the sodium salt of the phenoxyalkanoic acid or anti-inflammatory carboxylic acids, in methanol e.g

$$10\ NiCl_2\ 6H_2O + 5\ H_3shi + 3\ Hpko + 18\ NaOH + 2NaL \longrightarrow$$

$$[NiL]_2[12\text{-}MC_{Ni(II)N(shi)_2(pko)_2}\text{-}4][12\text{-}MC_{Ni(II)N(shi)_3(pko)}\text{-}4](MeOH)_3(H_2O) + 18H_2O + 20NaCl$$

The compounds are red-brown crystalline solid that appear to be air and moisture stable and insoluble in water. All metallacrowns are soluble in methanol, *$CHCl_3$, DMF, DMSO* and there are no electrolytes in these solvents.

### Structural features of the metallacrowns

A series of mixed ligand compounds with unique characteristics have been obtained using 2-dipyridyl-ketonoxime in conjunction with salicylhydroxamic acid. *Hpko* is a bifunctional ligand that can bind metals in either five or six membered chelate rings (scheme 2). The ligand can be singly deprotonated when metals are bound. The deprotonated di-2-pyridyl-ketonoxime uses ketonoxime oxygen ($O_K$) and one pyridine-nitrogen (N) to bind to one nickel and the other pyridine-nitrogen (N) plus ketonoxime nitrogen ($N_K$) to chelate an adjacent Ni(II). The deprotonated salicylhydroxamic acid acts as a binucleating ligand with the carbonyl and hydroxamate oxygens ($O_C$ and $O_H$) binding to one nickel and the phenolate oxygen ($O_{Ph}$) plus imine nitrogen (N) chelating an adjacent Ni(II).

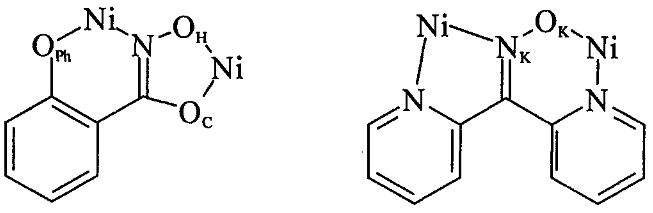

*shi* *pko*

**Scheme 2.** Drawings showing the binding modes of $shi^{3-}$ and $pko^{1-}$ with metal ions.

2:2 distribution of $shi^{3-}$ and $pko^{1-}$ results in a trinuclear $Ni_3(shi)_2(Hpko)_2(py)_2$ compound /44/ or in a tetranuclear $[12\text{-}MC_{Ni(II)N(Hshi)_2(pko)_2}\text{-}4]$ metallacrown ring [11]. When Ni(II) is captured in the center, the divalent pentanuclear $[Ni(II)(A)_2][12\text{-}MC_{Ni(II)N(shi)_2(pko)_2}\text{-}4]$ [A=carboxylato ligand] complex results /11/.

The stoichiometry of $shi^{3-}$ and $pko^{-1}$ 3:1 leads to the formation of a decanuclear metallacrown complex with the formula $[Ni(II)(A)]_2[12\text{-}MC_{Ni(II)N(shi)_2(pko)_2}\text{-}4][12\text{-}MC_{Ni(II)N(shi)_3(pko)}\text{-}4]$. This neutral decanuclear $[Ni(II)(A)]_2[12\text{-}MC_{Ni(II)N(shi)_2(pko)_2}\text{-}4][12\text{-}MC_{Ni(II)N(shi)_3(pko)}\text{-}4]$ fused metallacrown consists of two $[12\text{-}MC_{M(ox)N(ligand)}\text{-}4]$ units, the $\{Ni(II)(A)[12\text{-}MC_{Ni(II)N(shi)_2(pko)_2}\text{-}4]\}$ and $\{Ni(II)(A)[12\text{-}MC_{Ni(II)N(shi)_3(pko)}\text{-}4]\}$ with $1^+$ and $1^-$ charge, respectively (Figure 1). Each metallacrown unit has four ring Ni(II) ions and one additional encapsulated Ni(II) ion in planar arrangement. The anionic unit is bonded with cationic one creating binuclear moieties (Figure 2a). The $\{Ni(II)(A)[12\text{-}MC_{Ni(II)N(shi)_2(pko)_2}\text{-}4]\}$ cation with 1+ charge has an alternating pattern of $shi^{3-}$ and $pko^{1-}$ ligands as one cycles around the 12-MC-4 structure (Figure 1a). Three Ni(II) ions have octahedral configuration and the fourth one a square planar. The $\{Ni(II)(A)[12\text{-}MC_{Ni(II)N(shi)_3(pko)}\text{-}4]\}$ anion with three $shi^{3-}$ and one $pko^{1-}$ ligands forms an anionic metallacrown ring with $1^-$ overall charge (Figure 1b). The two metallacrown cores are formed through a $[Ni(II)\text{-}N\text{-}O\text{-}]_4$ repeat unit. Three Ni(II) ions are in octahedral and one in square planar environment analogous to the cationic unit. The cavity radii, 0.68 Å and 0.70 Å, for the anionic and cationic units of compound 3 /11,17/ respectively, allows encapsulation of the fifth nickel ion. (Figure 2b). The captured Ni(II) ion lies in the plane of the four ring-oxygen atoms. The herbicide or antiiflammatory carboxylato ligands are bridging the central octahedral nickel atom with a ring metal ion in a bindetate fashion (Figure 2b).

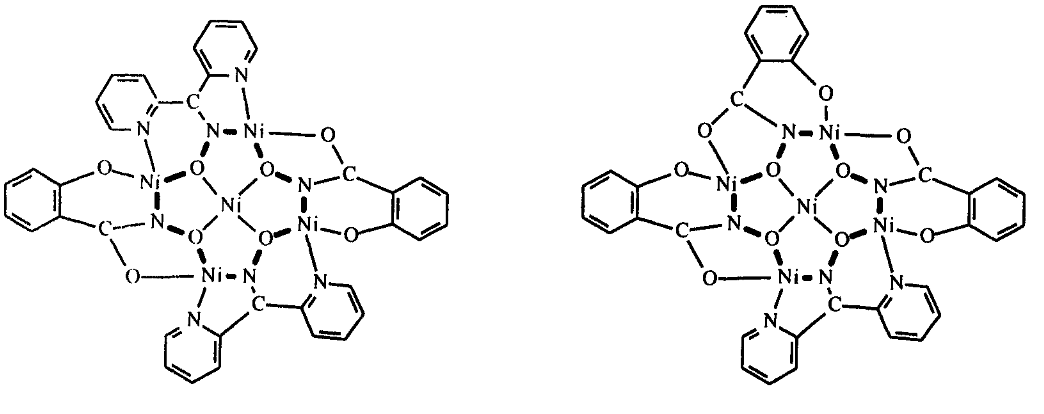

a b

**Fig. 1:** Drawings showing the connectivity pattern of the fused dimer $[Ni(II)(\textit{carboxylato})]_2[12\text{-}MC_{Ni(II)N(\textit{shi})_2(\textit{pko})_2}\text{-}4][12\text{-}MC_{Ni(II)N(\textit{shi})_3(\textit{pko})}\text{-}4](CH_3OH)_3\ (H_2O)$

a b

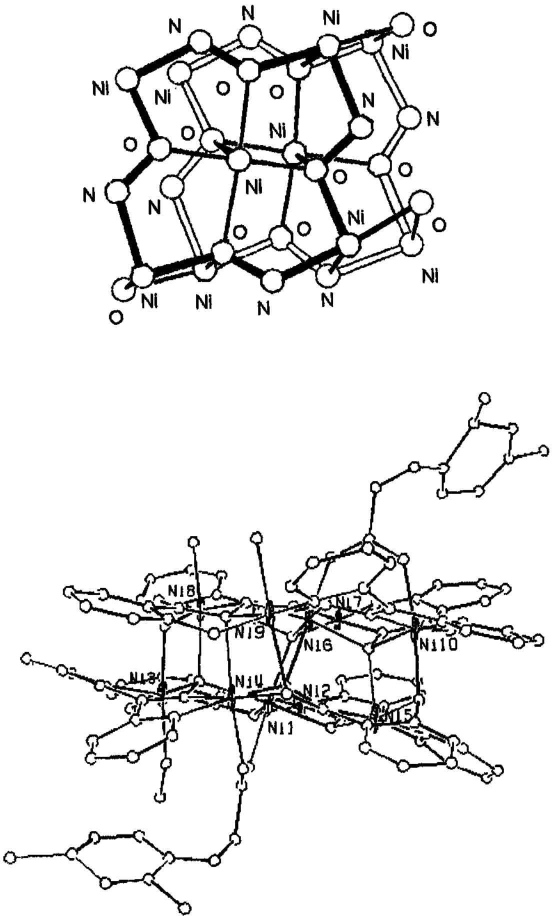

**Fig. 2:** a) Drawing showing the interaction of the metallacrown rings. b) An ORTEP view of the structure of the compound 4 emphasizing the interaction of the two metallacrown rings

### Spectroscopic study

All the metallacrowns show strong bands about 1600, 1480, 1465 and 1265 $cm^{-1}$ assigned to $\nu(C{=}N)_{shi,pko}$, $\nu(C{=}O_{ph})_{shi}$, $\nu(C{=}O_{ox})_{shi}$ and $\nu$ $(N\text{-}O_{ox})_{shi,pko}$ stretching frequencies respectively, while the presence of $\nu_{asym}(CO_2)$ and $\nu_{sym}(CO_2)$ bands at about 1570 and 1440 $cm^{-1}$ with $\Delta \approx 130$ $cm^{-1}$ support a double-bridging mode of binding of the carboxylato ligands. The IR patterns of all the metallacrowns are very similar suggesting structural similarities. The solution paramagnetic $^1H$ NMR spectra of the metallacrowns with *ibuprofen* and *naproxen* as accommodated ligands, compounds **5** and **6**, show six distinct downfield resonances in a range from 40 to 10 ppm. The downfield pattern is similar for all the metallacrowns allowing these resonances to be attributed to the protons of the metallacrown rings. In Schiff-base Ni(II)-complexes, the protons were found to be downfield shifted with the –CH=N group to be the most paramagnetically shifted signal, at about 300 ppm /45,46/; that is not the case for our compounds as most of the protons are quite apart from the paramagnetic centers. One would expect up to twelve resonances for the metallacrown part of the complexes, eight from *pko* and four from *shi* and 4-8 resonances from the bound carboxylato ligands. A complete assignment of resonances was not accomplished because selective deuteration of the *pko* and carboxylato ligands is an arduous task. FAB Mass Spectroscopy was used to determine the molecular weight of the species in solution. Almost all the compounds give a molecular weight fragment at FAB-MS (e.g. compound **6**, Figure 3) suggesting that the compounds keep their integrity in solution.

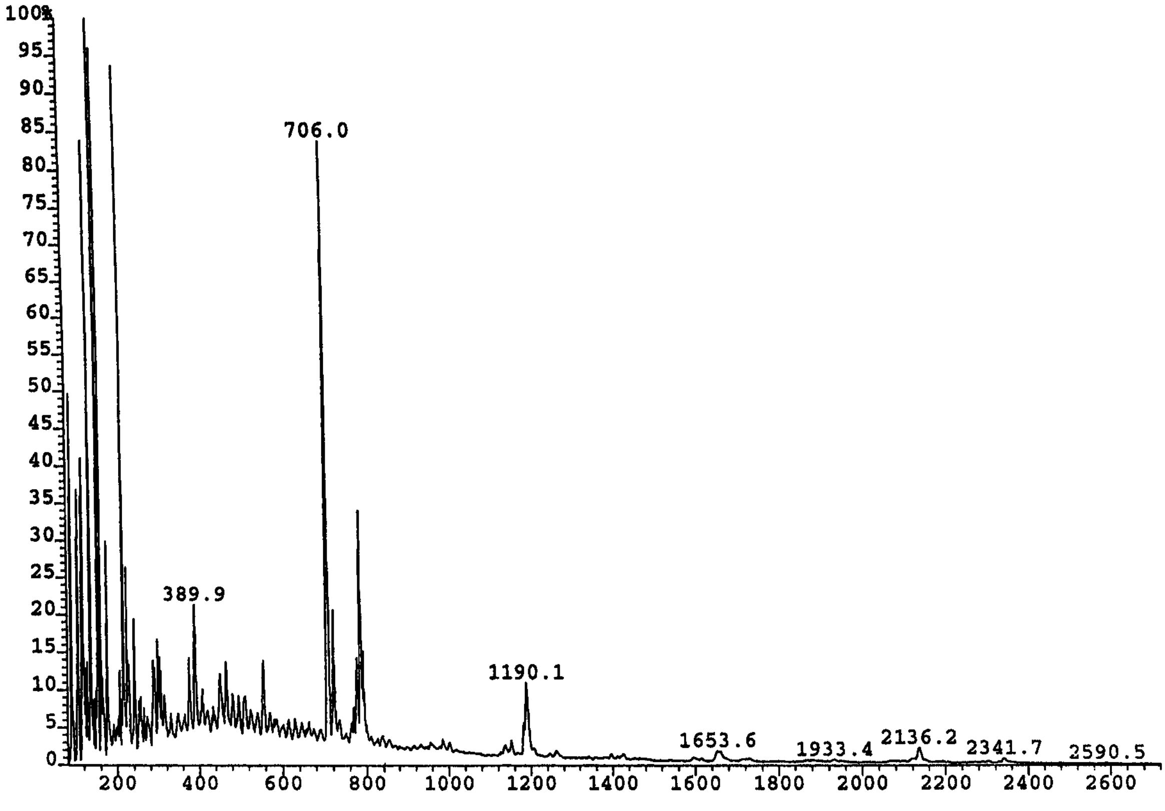

**Fig. 3:** A FAB-MS spectrum of the compound **6**

### *In vitro* effects of nickel compounds on DNA

The effect of the newly synthesized complexes of Ni(II) with high metal nuclearity, on the integrity and electrophoretic mobility of nucleic acids was examined. Figure 4 shows that compounds **1, 2, 4** and **5** (lanes 1, 2, 4 and 5, respectively), at concentration 1.2 mM affect the electrophoretic mobility of supercoiled and relaxed pDNA, whereas compounds **3, 6** do not show any effect. The effect of compound **1** becomes more profound in the experiment of **Figure 5**, where increasing concentrations of the compound **1** were used. Retardation in the electrophoretic mobility of supercoiled and relaxed pDNA occurred or precipitates in the top of the gel at higher concentrations of compound **1**. The changes on the mobility can be attributed to the altered structures of the pDNA treated with Ni(II) complexes. Compound **1** was effective even at the concentration of 0.4 mM. The effect of ligands or the solvent (DMSO) on the pDNA mobility was tested as

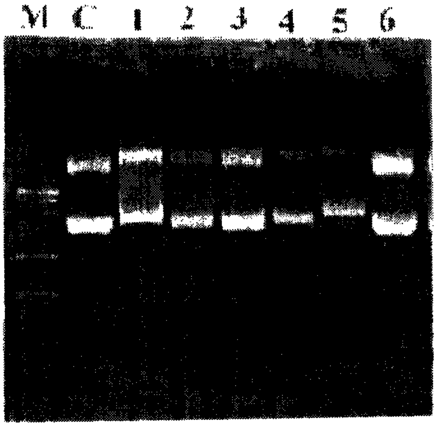

**Fig. 4:** Agarose (1%) gel electrophoresis pattern of pUC19 DNA treated with compounds **1-6**. Two μg of pUC19 DNA incubated at 37°C for 1h. Lane C: control (pUC19 DNA not treated with compounds). Lane 1-6: pUC19 DNA treated with 1.2 mM of the compounds **1-6**. Lane M: Molecular weight markers, 1 Kb DNA ladder: 10.0, 8.0, 6.0, 5.0, 4.0, 3.0, 2.0, 1.5 and 1.0.

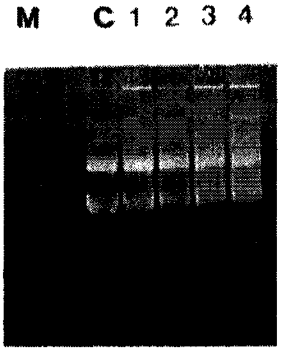

**Fig. 5:** Agarose (1%) gel electrophoresis pattern of pUC19 DNA treated with increasing concentrations of compound **1**. Two μg of pUC19 DNA incubated at 37°C for 1h. Lane C: control (pUC19 DNA not treated with compounds). Lane 1-4: pUC19 DNA treated with 0.4, 0.8, 1.2, 1.6 mM of the compound **1**. Lane M: Molecular weight markers, 1 Kb DNA ladder: 10.0, 8.0, 6.0, 5.0, 4.0, 3.0, 2.0, 1.5 and 1.0.

well, with no remarkable effect. Similar results with the compound **1** were observed when linearized plasmid DNA, ds or ssDNA were used (data not shown).

### Antibacterial study of nickel compounds

The results of antibacterial screening data, for the nickel compounds tested, on *B. subtilis, B. cereus, S. aureus* (Gram positive), and *X. campestris, E. coli, P. mirabilis* (Gram negative), are shown in Table 1. Among all the compounds tested, the most active was compound **1**. The best M.I.C value (12 μg/ml) was presented by compound **1**, with the most sensitive microorganism the *B. subtilis*. The effect of all substitutes was tested separately on all cultures without causing any growth effect.

Evaluating the above data by comparing the antibacterial activity of the compounds tested and the effect on DNA we can conclude that complexes that exhibit antibacterial activity were those that caused alterations on DNA. However, a correlation between DNA interference and antibacterial properties of the new compounds must be further elucidated.

**Table 1**

Antibacterial study of the nickel compounds **1-6**.

| | **Gram (-) bacteria** | | | **Gram (+) bacteria** | | |
|---|---|---|---|---|---|---|
| * | X. campestris (μg/ml) | *E. coli* (μg/ml) | *P. mirabilis* (μg/ml) | *B. subtilis* (μg/ml) | *B. cereus* (μg/ml) | *S. aureus* (μg/ml) |
| **1** | 50 | 25-50 | 50-100 | 12-25 | 25 | 25 |
| **2** | ~50 | >100 | >100 | 50 | 50 | 50 |
| **3** | 100 | >100 | >100 | 50 | ~50 | 100 |
| **4** | ~50 | 100 | >100 | 50 | ~50 | 100 |
| **5** | 50 | 50-100 | >100 | ~25 | ~25 | 50 |
| **6** | ~100 | 100 | >100 | 50 | 100 | 100 |

* **1** $[Ni(2,4\text{-}DP)]_2[12\text{-}MC_{Ni(II)N(shi)_2(pko)_2}\text{-}4][12\text{-}MC_{Ni(II)N(shi)_3(pko)}\text{-}4](MeOH)_3(H_2O)$

**2** $[Ni(2,4,5\text{-}T)]_2[12\text{-}MC_{Ni(II)N(shi)_2(pko)_2}\text{-}4][12\text{-}MC_{Ni(II)N(shi)_3(pko)}\text{-}4](MeOH)_3(H_2O)$

**3** $[Ni(MCPA)]_2[12\text{-}MC_{Ni(II)N(shi)_2(pko)_2}\text{-}4]\ [12\text{-}MC_{Ni(II)N(shi)_3(pko)}\text{-}4](MeOH)_3(H_2O)$

**4** $[Ni(2,4\text{-}D)]_2[12\text{-}MC_{Ni(II)N(shi)_2(pko)_2}\text{-}4][12\text{-}MC_{Ni(II)N(shi)_3(pko)}\text{-}4](MeOH)_3(H_2O)$

**5** $[Ni(naproxen)]_2[12\text{-}MC_{Ni(II)N(shi)_2(pko)_2}\text{-}4]\ [12\text{-}MC_{Ni(II)N(shi)_3(pko)}\text{-}4](MeOH)_3(H_2O)$

**6** $[Ni(ibuprofen)]_2[12\text{-}MC_{Ni(II)N(shi)_2(pko)_2}\text{-}4]\ [12\text{-}MC_{Ni(II)N(shi)_3(pko)}\text{-}4](MeOH)_3(H_2O)$

**ACKNOWLEDGMENT.**

This work was carried out within the framework of EPEAEK – "Bioinorganic Chemistry".

**REFERENCES**

1. V.L. Pecoraro, A.J. Stemmler, B.R. Gibney, J.J. Bodwin, H. Wang, J.W. Kampf and A. Barwinski, in: K.D. Karlin (Ed.) *Progress in Inorganic Chemistry*, Vol **45**, John Wiley & Sons Inc., New York, (1997) p. 83.
2. V.L. Pecoraro, *Inorg. Chim. Acta*, **155**, 171 (1989)
3. M.S. Lah, M.L. Kirk, W. Hatfield and V.L. Pecoraro, *J. Chem. Soc. Chem. Commun.*, 1606 (1989)
4. B.R. Gibney, A.J. Stemmler, S. Pilotek, J.W. Kampf and V.L. Pecoraro, *Inorg. Chem.*, **32**, 6008 (1993)
5. M.S. Lah and V. L. Pecoraro, *J. Am. Chem. Soc.*, **111**, 7258 (1989)
6. M.S. Lah and V. L. Pecoraro, Comm. *Inorg. Chem.*, **11**, 59 (1990)
7. A.J. Stemmler, J.W. Kampf, M.L. Kirk and V.L. Pecoraro, *J. Am. Chem. Soc.*, **117**, 6368 (1995)
8. B.R. Gibney, H. Wang, J.W. Kampf and V.L. Pecoraro, *Inorg. Chem. 35*, 6184 (1996)
9. M.S. Lah and V.L. Pecoraro, *Inorg. Chem.*, **30**, 878 (1991)
10. B.R. Gibney, D.P. Kessissoglou, J.W. Kampf and V.L. Pecoraro, *Inorg. Chem.*, **33**, 4840 (1994)
11. G. Psomas, A. J. Stemmler, C. Dendrinou-Samara, J. Bodwin, M. Schneider, M. Alexiou, J.W. Kampf, D. P. Kessissoglou and V.L. Pecoraro, *Inorg. Chem.* **40**, 1562 (2001)
12. D.P. Kessissoglou, J. Bodwin, J.W. Kampf, C. Dendrinou-Samara and V.L. Pecoraro, *Inorg. Chim. Acta*, **331**, 73 (2002)
13. D.P. Kessissoglou, J.W. Kampf and V.L. Pecoraro, *Polyhedron*, **13**, 1379 (1994)
14. A.J. Stemmler, A. Barwinski, M.J. Baldwin, V. Young and V.L. Pecoraro, *J. Am. Chem. Soc.*, **118**, 11962 (1996)
15. A.J. Stemmler, J.W. Kampf and V.L. Pecoraro, *Angew. Chem. Int. Ed. Engl.*, **35**, 2841 (1996)
16. A.J. Stemmler, J.W. Kampf, M.L. Kirk and V.L. Pecoraro, *Inorg. Chem.*, **34**, 2271 (1995)
17. C. Dendrinou-Samara, G. Psomas, L. Iordanidis, V. Tangoulis and D.P. Kessissoglou, *Chem. Eur. J.*, **7**, 5041 (2001)
18. C. Dendrinou-Samara, L. Alevizopoulou, L. Iordanidis, E. Samaras and D.P. Kessissoglou, *J. Inorg. Biochem.*, **89**, 89 (2002)
19. B. Kwak, H. Rhee, S. Park and M.S. Lah, *Inorg. Chem.*, **37**, 3599 (1998)
20. S.-X. Liu, S. Lin, B.-Z. Lin, C.-C. Lin and J.-Q. Huang, *Angew. Chem. Int. Ed. Engl.*, **40**, 1084 (2001)
21. R.W. Saalfrank, R. Burak, S. Reihs, N. Löw, F. Hampel, H.-D. Stachel, J. Lentmaier, K. Peters, E.-M. Peters and H. G. von Schering, *Angew. Chem. Int. Ed. Engl.*, **34**, 993 (1995)
22. R.W. Saalfrank, I. Bernt, E. Uller and F. Hampel, *Angew. Chem. Int. Ed. Engl.*, **36**, 2482 (1997)
23. R.W. Saalfrank, N. Löw, S. Kareth, V. Seitz, F. Hampel, D. Stalke and M. Teichert, *Angew. Chem. Int. Ed. Engl.*, **37**, 172 (1998)

24. G. Psomas, C. Dendrinou-Samara, M. Alexiou, A. Tsohos, C.P. Raptopoulou, A. Terzis and D.P. Kessissoglou, *Inorg. Chem.*, **37**, 6556 (1998)
25. M.S. Lah, B.R. Gibney, D.L. Tierney, J.E. Penner-Hahn and V.L. Pecoraro, *J. Am. Chem. Soc.*, **115**, 5857 (1993)
26. R.W. Saalfrank, I. Bernt, M.M. Chowdhry, F. Hampel and G.B.M. Vaughan, *Chem. Eur. J.*, **7**, 2765 (2001)
27. M. Eshel, A. Bino,·I. Feiner, D.C. Johnston, M. Luban and L.L. Miller, *Inorg. Chem.*, **39**, 1376 (2000)
28. K.S.Kasprzak, B.Diwan, N. Konishi, M. Misra and J.M. Rice, *Carcinogenesis* **11**, 647(1990)
29. M. Nishimura and M. Umeda, *Mutation Res.*, **68**, 337 (1979)
30. M.L. Larramendy, N.C. Popescu and.J.A. Dipaolo, *Environ Mutat.*, **3**, 595 (1981)
31. P. Sen and M. Costa, *Cancer Res.*, **45**, 2320 (1985)
32. A. Hartwing, *Toxicology Letters* **102-103**, 235 (1998)
33. T.P. Coogan, D.M. Latta, E.T. Snow and M. Costa, *CRC Crit. Rev. Toxicol.*, **19**, 341 (1989)
34. V.A. Sorokin, G.O. Vallev, G.O. Gladchenko, I.V. Sysa, Y.P. Blagoi and I.V. Volchok, *J. Inorg. Biochem.*, **63**, 99 (1996)
35. A.K. Datta, C.W. Riggs, Jr.M.J. Fivash and K.S. Kasprzak, *Chem.–Biol. Interact.*, **79**, 323 (1991)
36. C. Dendrinou-Samara, D. P. Kessissoglou, G. E. Manoussakis, D. Mentzafos and A. Terzis, *J. Chem. Soc., Dalton Trans.* 959 (1990).
37. C. Dendrinou-Samara, P. D. Jannakoudakis, D. P. Kessissoglou, G. E. Manoussakis, D. Mentzafos and A. Terzis, *J. Chem. Soc., Dalton Trans.* 3259 (1992).
38. C. Dendrinou-Samara, G. Psomas, K. Christophorou, V. Tangoulis, V. P. Raptopoulou, A. Terzis and D. P. Kessissoglou, *J. Chem. Soc., Dalton Trans.* 3737 (1996).
39. G. Psomas, C. Dendrinou-Samara, P. Philippakopoulos, V. Tangoulis, C. P. Raptopoulou, H. Samaras and D. P. Kessissoglou, *Inorg. Chim. Acta* **272**, 24 (1998).
40. C. Dendrinou-Samara, G. Tsotsou, C. P. Raptopoulou, A. Kortsaris, D. Kyriakidis and D. P. Kessissoglou, *J. Inorg. Biochem.* **71**, 171 (1998)
41. G. Psomas, C. P. Raptopoulou, L. Iordanidis, C. Dendrinou-Samara, V. Tangoulis and D. P. Kessissoglou, *Inorg. Chem.* 39, 3042 (2000).
42. C. Dendrinou-Samara, G. Psomas, C. P. Raptopoulou and D. P. Kessissoglou, *J. Inorg. Biochem.*, **83**, 7 (2001)
43. C. Dendrinou-Samara, L. Alevizopoulou, L. Iordanidis, E. Samaras and D. P. Kessissoglou. *J. Inorg. Biochem.* **89**, 89 (2002).
44. M. Alexiou, I. Tsivikas, C. Dendrinou-Samara, A.A. Pantazaki, P.Trikalitis, N. Lalioti, D.A. Kyriakidis and D.P. Kessissoglou, *J. Inorg. Biochem.* 00 (2003).
45. I. Bertini and C. Luchinat, In: *NMR of Paramagnetic Molecules in Biological Systems;* Benjamin/Cummings : Menlo Park, CA, 1986.
46. I. Bertini and C. Luchinat, *Coord. Chem. Rev.* 150 (1996).

